# Coenzyme q10 may attenuate cyclophosphamide-induced renal damage

**DOI:** 10.1590/1806-9282.20240772

**Published:** 2024-08-30

**Authors:** Daoyun Lei

**Affiliations:** 1 Zhongda Hospital Southeast University (jiangbei), Department of Anesthesiology – Nanjing, China.

Dear Editor,

We read an interesting article recently published by Kara^1^. The article revealed that coenzyme q10 may be a potential therapeutic option for preventing cyclophosphamide-induced kidney injury. The therapeutic efficacy of coenzyme q10 was assessed using a kidney injury model established with cyclophosphamide and in the setting of right renalectomy. Biochemical assays such as blood malondialdehyde levels and catalase and superoxide dismutase activities were measured. To further define the renal histopathological damage, a histopathological examination was used to assess the renal histopathological damage. The study showed that tissue damage was significantly higher in the cyclophosphamide group than in the cyclophosphamide+coenzyme q10 group. Biochemical results also supported similar results. The results of this study provide evidence that coenzyme q10 is an effective treatment for the prevention or treatment of cyclophosphamide-induced renal injury. This provides a therapeutic direction for treating cyclophosphamide-induced kidney injury, but more clinical studies are needed to prove it. However, I believe more factors need to be considered to promote the protective effect of coenzyme q10 against cyclophosphamide-induced kidney injury.

First, cyclophosphamide is currently used in combination chemotherapy regimens with other anticancer drugs to treat many tumor diseases. However, it is worth noting that it has some toxic effects on the heart and kidneys, especially at high doses (>120 mg/kg). The authors used a dose of 30 mg/kg, which is low compared to the dose required for kidney damage. Undeniably, despite the high degree of similarity between rats and humans, the renal damage caused by different concentrations of cyclophosphamide still requires further study. However, cyclophosphamide can be used as an immunosuppressant in various autoimmune diseases, especially nephrotic syndrome. This is an immune-related disease in which the body produces antibodies against itself that attack the kidneys, increasing the permeability of the kidney’s filtration membrane and causing a large amount of protein loss. Therefore, cyclophosphamide is a drug that can both damage kidney function and treat kidney disease. Kidney damage caused by cyclophosphamide must be elaborated on from a different perspective, considering its renal therapeutic effects.

Second, the author’s assertion of coenzyme q10’s promising therapeutic efficacy in treating cyclophosphamide-induced renal injury is noteworthy. However, it is crucial to acknowledge the potential side effects of coenzyme q10, such as gastrointestinal symptoms (nausea, vomiting, loss of appetite, and abdominal pain), which have yet to be thoroughly explored. Given the widespread use of cyclophosphamide in cancer treatment, it is important to note that the American Cancer Society has raised concerns about coenzyme q10 potentially reducing the effectiveness of chemotherapy and radiation therapy. Consequently, most oncologists advise against its use during cancer treatment. To provide a more comprehensive understanding, the author could consider discussing the therapeutic effects of coenzyme q10 in the context of a specific disease situation beyond just cyclophosphamide-induced renal injury.

Overall, the author describes the therapeutic effect of coenzyme q10 on cyclophosphamide-induced renal injury, which is instructive for clinical treatment. However, more and better research is needed to demonstrate this.
